# Magnetic resonance imaging of arterial stroke mimics: a pictorial review

**DOI:** 10.1007/s13244-018-0637-y

**Published:** 2018-06-22

**Authors:** Gilles Adam, Marine Ferrier, Sofia Patsoura, Raluca Gramada, Zuzana Meluchova, Vanessa Cazzola, Jean Darcourt, Christophe Cognard, Alain Viguier, Fabrice Bonneville

**Affiliations:** 1 Department of Diagnostic and Therapeutic Neuroradiology, Hôpital Purpan, Place du Docteur Baylac, TSA 40031, 31059 Toulouse, France; 2 Department of Neurology, Hôpital Purpan, Place du Docteur Baylac, TSA 40031, 31059 Toulouse, France

**Keywords:** Acute ischaemic stroke, Stroke mimics, Stroke diagnosis, Diffusion-weighted imaging, Magnetic resonance imaging

## Abstract

**Abstract:**

Acute ischaemic stroke represents the most common cause of new sudden neurological deficit, but other diseases mimicking stroke happen in about one-third of the cases. Magnetic resonance imaging (MRI) is the best technique to identify those ‘stroke mimics’. In this article, we propose a diagnostic approach of those stroke mimics on MRI according to an algorithm based on diffusion-weighted imaging (DWI), which can be abnormal or normal, followed by the results of other common additional MRI sequences, such as T2 with gradient recalled echo weighted imaging (T2-GRE) and fluid-attenuated inversion recovery (FLAIR). Analysis of the signal intensity of the parenchyma, the intracranial arteries and, overall, of the veins, is crucial on T2-GRE, while anatomic distribution of the parenchymal lesions is essential on FLAIR. Among stroke mimics with abnormal DWI, T2-GRE demonstrates obvious abnormalities in case of intracerebral haemorrhage or cerebral amyloid angiopathy, but this sequence also allows to propose alternative diagnoses when DWI is negative, such as in migraine aura or headaches with associated neurological deficits and lymphocytosis (HaNDL), in which cortical venous prominence is observed at the acute phase on T2-GRE. FLAIR is also of major interest when DWI is positive by better showing evocative distribution of cerebral lesions in case of seizure (involving the hippocampus, pulvinar and cortex), hypoglycaemia (bilateral lesions in the posterior limb of the internal capsules, corona radiata, striata or splenium of the corpus callosum) or in posterior reversible encephalopathy syndrome (PRES). Other real stroke mimics such as mitochondrial myopathy, encephalopathy, lactic acidosis, stroke-like episodes (MELAS), Susac’s syndrome, brain tumour, demyelinating diseases and herpes simplex encephalitis are also included in our detailed and practical algorithm.

**Key points:**

*• About 30% of sudden neurological deficits are due to non-ischaemic causes.*

*• MRI is the best technique to identify stroke mimics.*

*• Our practical illustrated algorithm based on DWI helps to recognise stroke mimics.*

## Introduction

Magnetic resonance imaging (MRI) is the best way to detect early signs of cerebral ischaemia and intracranial haemorrhage [1]. Rapid MRI sequences reduce the length of the investigation, giving valuable information with an acceptable time in terms of patient care. For this reason, MRI is increasingly used to select candidates for thrombolysis. After a few minutes, acute arterial ischaemic stroke (AIS) appears as hyperintense on diffusion-weighted imaging (DWI), with a corresponding reduction of the apparent diffusion coefficient (ADC). However, DWI may also be negative at the very beginning of an ischaemic stroke, as it requires several tens of minutes for the cytotoxic oedema to return hyperintensity.

If AIS represents the most common cause of new sudden neurological deficit, other diseases mimicking ischaemic strokes represent up to one-third of cases [2] and are termed ‘stroke mimics’. It is important to promptly identify these differential diagnoses to avoid inappropriate urgent treatment, but also to prevent inadequate long-term prevention treatment. Though computed tomography (CT) scanning is the standard technique in many stroke centres around the world and has demonstrated its effectiveness in the decision-making process for the treatment of AIS, MRI with DWI has a better sensitivity to depict small acute ischaemic cerebral lesions, but also offers the possibility, at the same time, to identify differential diagnoses to stroke in case of a sudden neurological deficit.

**Fig. 1:**
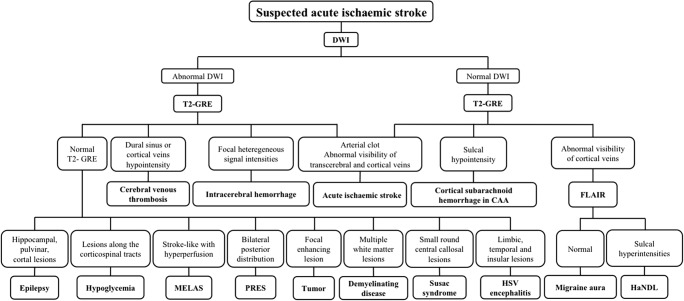
Algorithm of stroke mimics diagnostic based on diffusion-weighted imaging (DWI) and common additional magnetic resonance imaging (MRI) sequences

Therefore, when performing MRI when faced with an acute neurological deficit, radiologists should be able to: (1) exclude haematoma, (2) confirm the positive diagnosis of AIS and (3) exclude ‘stroke mimics’. In this review, we will recall the expected results of a simple magnetic resonance protocol in the case of AIS. Then, we will propose a practical illustrated algorithm of the main ‘stroke mimics’ based on the results of DWI. As previously mentioned, because DWI can be positive or negative in the case of AIS, stroke mimics can be dichotomised in the same way. Common additional magnetic resonance sequences then provide enough data to evoke substitute diagnoses (Fig. 1).

## Acute ischaemic stroke

MRI has significantly higher sensitivity and specificity than CT in the diagnosis of acute ischaemic stroke in the first few hours after onset, especially with the use of DWI. DWI is, indeed, able to detect ischaemic changes within minutes after onset. It is true, however, that a stroke may appear normal on DWI if it is very acute or may be seen beyond the first minutes. Patients without a lesion are more likely to be women or have previous stroke [3]. Reduced proton motion due to cytotoxic oedema explains the reduction of the ADC, which appears with high signal intensity on DWI. Lesions are distributed to arterial territories, with a predilection for grey matter when very acute.

Though T2-weighted images (T2-WI) and fluid-attenuated inversion recovery (FLAIR) sequences are less sensitive than DWI in demonstrating parenchymal changes in the first few hours after stroke onset, loss of arterial signal void distal to a large artery occlusion may be observed immediately. Such FLAIR intravascular hyperintensities in the subarachnoid spaces are associated with diffusion–perfusion mismatch [4].

FLAIR sequences can also be used as a clock as part of a DWI–FLAIR mismatch: when lesions are hyperintense on DWI but not on FLAIR, this is considered as a reliable MRI, signalling a stroke onset of under 4.5 h [5].

T2 with gradient recalled echo weighted imaging (T2-GRE) and susceptibility-weighted imaging (SWI) are highly sensitive, and can detect thrombus occlusions in intracranial arteries. Through blooming thanks to a magnetic susceptibility artefact described as the ‘susceptibility vessel sign’, clots may be depicted, located and measured with precise assessment [6]. Together with time-of-flight magnetic resonance angiography (TOF-MRA), T2-GRE and SWI may identify the site of the occlusion and adjust treatment planning and patient care, as proximal occlusion and clots longer than 8 mm are associated with a low recanalisation rate after IV thrombolysis [7]. It is, therefore, of major importance to characterise the thrombus, especially if endovascular clot retrieval is discussed. It has also been reported that T2-GRE and SWI help identify hypoperfused regions downstream of a cerebral artery occlusion, by demonstrating abnormal visibility of cortical and deep veins draining such territories.

During the acute phase of ischaemic stroke, perfusion-weighted imaging (PWI) reveals reductions in cerebral blood flow (CBF) in hypoperfused areas of brain parenchyma. Autoregulation attempts to preserve CBF values by inducing vasodilatation, which results in an increased cerebral blood volume (CBV). Brain regions characterised by reduced CBF and increased CBV are considered areas of ischaemic penumbra. Conversely, when autoregulation is unable to maintain the CBV, that is to say, when both CBF and CBV are reduced, these brain regions represent the infarct core, where tissue that is no longer viable will not benefit from reperfusion. A popular paradigm is that patients with acute ischaemic stroke might benefit from recanalisation if significant penumbra can be shown by MRI. However, there is still a lack of consensus regarding the best definition and optimal measurement of the diffusion–perfusion mismatch [8].

To summarise, territorial acute ischaemic stroke appears most of the time with hyperintensity on DWI and reduced ADC values, downstream of a clot, demonstrating low T2-GRE signal intensity, occluding an artery, as observed on TOF-MRA. Hyperintensity on FLAIR classically appears after 4.5 h. PWI makes it possible to distinguish ischaemic penumbra from the infarct core.

If contrast-enhanced T1-WI were to be performed after PWI, it would never show parenchymal enhancement in the first few hours of stroke onset. If such enhancement was observed, an alternative diagnosis—a stroke mimic—should be considered.

## Stroke mimics with abnormal DWI

### Intracerebral haemorrhage

Intracerebral haemorrhages account for 15% of strokes [9] and may be revealed by a sudden neurological deficit. Elderly people are the most affected population [10]. At this age, high blood pressure is the main cause of spontaneous brain haematomas, which are mainly located in the basal ganglia and the internal capsules. Other sites of hypertensive haemorrhaging are the pons and the cerebellum. Lobar haemorrhages are more often associated with cerebral amyloid angiopathy in the elderly. In the younger population, the presence of aneurysms or arteriovenous malformation (AVM) ruptures, tumours, infections or coagulopathies must be verified.

The appearance of the haematoma on MRI depends on its age and size. In hyperacute haemorrhages (intracellular oxyhaemoglobin), DWI shows hyperintensity with corresponding ADC restriction, thus mimicking ischaemic stroke. The heterogeneity of the signal intensity and other MRI sequences are used to rectify the diagnosis.

**Fig. 2:**
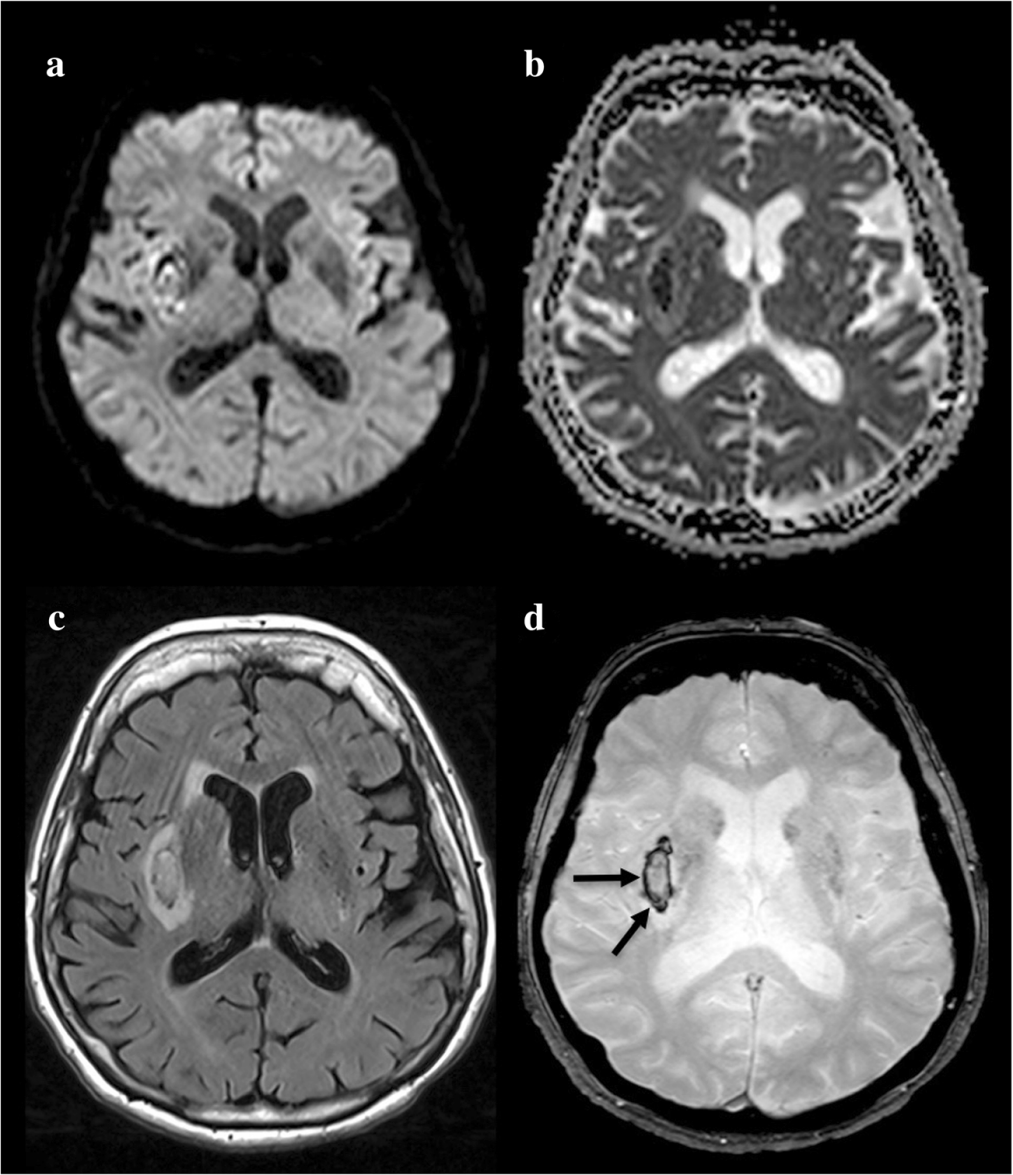
A 48-year-old woman with intracerebral haemorrhage presenting with left hemiplegia and left homonymous hemianopsia. DWI shows heterogeneous signal abnormalities involving the right lenticular nucleus on b1000 (**a**), apparent diffusion coefficient (ADC) (**b**) and fluid-attenuated inversion recovery (FLAIR) (**c**). The lesion appears as a peripheral hypointensity on T2 with gradient recalled echo weighted imaging (T2-GRE) (**d**, arrows)

Hyperacute haemorrhages are also isointense on T1-WI, iso- or hyperintense on T2-WI and demonstrate characteristic peripheral hypointensity on T2-GRE or SWI (Fig. 2). Areas of gadolinium leakage within acute haematomas, as observed on post-contrast T1-WI, also called the ‘spot sign’, are correlated with an increased risk of haematoma expansion [11].

### Cerebral venous thrombosis

Cerebral venous thrombosis is an important life-threatening neurological condition. It is more common in women, who represent approximately 75% of adult cases [12]. The diagnosis may be challenging in view of the range of clinical symptoms and the variability of the imaging findings, both potentially mimicking AIS.

When suspecting cerebral venous thrombosis, radiologists have a key role and must fulfil three goals: (1) confirm the diagnosis by showing direct signs of occlusion of the venous structure by a thrombus, (2) look for signs of venous infarction in the brain parenchyma and, finally, (3) try to find an origin or a pathology related to this thrombosis [13]. Venous ischaemia begins with a vasogenic oedema that can be accompanied by cytotoxic oedema [14]. Thus, DWI shows variable signal abnormalities within the same parenchymal lesion of venous ischaemia. The topography of such parenchymal changes lacks arterial distribution and depends on where the thrombus lies within the cerebral venous system. Restricted diffusion of venous ischaemia has no prognostic value [15]. These lesions appear hyperintense on T2-WI and FLAIR. T2-GRE may highlight frequent haemorrhagic components. Cerebral venous thrombosis may rarely present as isolated subarachnoid haemorrhage that is in the vicinity of the thrombus [16]. The signal intensity of the clot depends on its age.

**Fig. 3:**
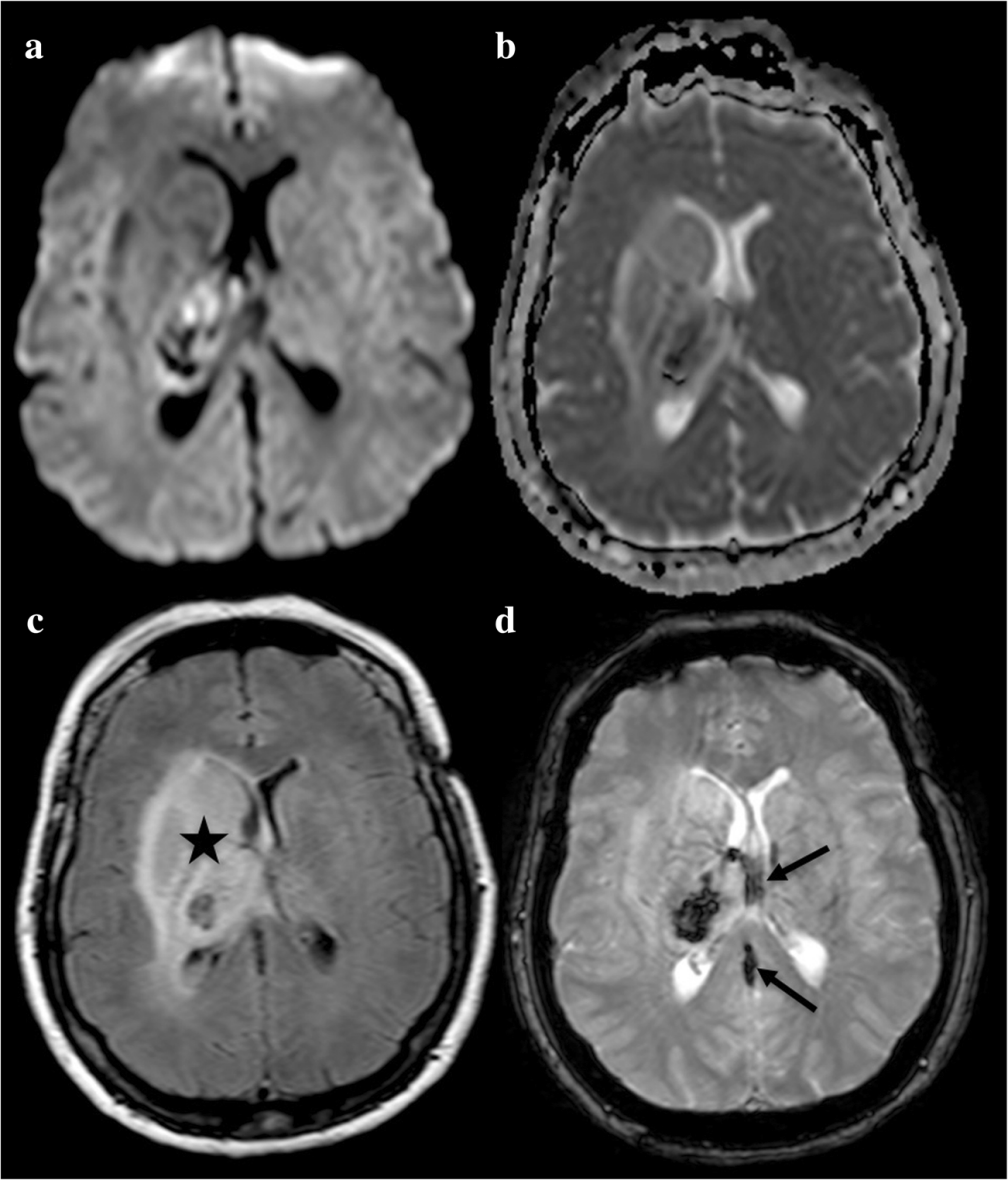
A 50-year-old woman with cerebral venous thrombosis presenting with drowsiness and left hemiplegia. DWI shows heterogeneous signal abnormalities on b1000 (**a**) and ADC (**b**). Hypersignal FLAIR swelling (**c**, star). T2-GRE shows an artefactual hypointensity in the right internal cerebral vein, the great vein of Galen and the straight sinus (**d**, arrows)

In the acute phase, during the very first days, the clot is barely visible on spin echo sequences and can be missed as it appears isointense to the brain parenchyma on T1-WI and hypointense on T2-WI and FLAIR [17], similarly to normal circulating veins. T2-GRE is the key sequence, because, due to the susceptibility artefact, it shows abnormal hypointensity of the thrombus blooming and enlarging the occluded venous structure (Fig. 3).

In the subacute phase (day 5 to day 15), the clot contains extracellular methaemoglobin and appears hyperintense on all sequences, i.e. T1-WI, T2-WI, FLAIR, but also T2-GRE and DWI [17].

In the chronic phase (after 15 days), the thrombus signal depends on the degree of organisation of the thrombus, but is typically isointense on T1-WI, iso- or hyperintense on T2-WI and hypointense on T2-GRE. The clot may enhance after a gadolinium injection. A clue to easily depict the thrombus in a dural sinus is to know that normal venous flow shows opposite signal intensity on FLAIR and T2-GRE. Therefore, in the case of cerebral venous thrombosis, occluded venous structures abnormally appear with identical signal intensities on these sequences, whatever the stage or the age of the thrombosis [18]. Venous occlusion should be visualised by magnetic resonance venography. Contrast-enhanced sequences are recommended to avoid flow artefacts. They show a filling defect of the occluded venous structure, showing the classic empty delta sign. This should not be confused with Paccioni granulations, which are typically focal, regular, well-defined and preferentially located along the superior sagittal sinus and close to the junction between the transverse and sigmoid sinus. Additional CT angiography should be performed in case of residual doubt, as this works remarkably well to demonstrate venous sinus thrombosis. Isolated cortical vein thrombosis is best seen with T2-GRE or, even better, SWI [19].

Finally, the radiologist needs to look for associated magnetic resonance features of an origin or a pathology related to this thrombosis, such as local infection, trauma, systemic diseases like Behcet’s disease, tumours or even intracranial hypotension.

### Epilepsy

Epilepsy is one of the most frequent stroke mimics. Some symptoms, such as headaches, involuntary movements, incontinence or postictal confusion, may be helpful pointers against stroke. However, seizures with partial features may be difficult to distinguish from real AIS, especially in the case of ‘negative’ symptoms, such as Todd’s paresis or postictal aphasia/dysphasia.

**Fig. 4:**
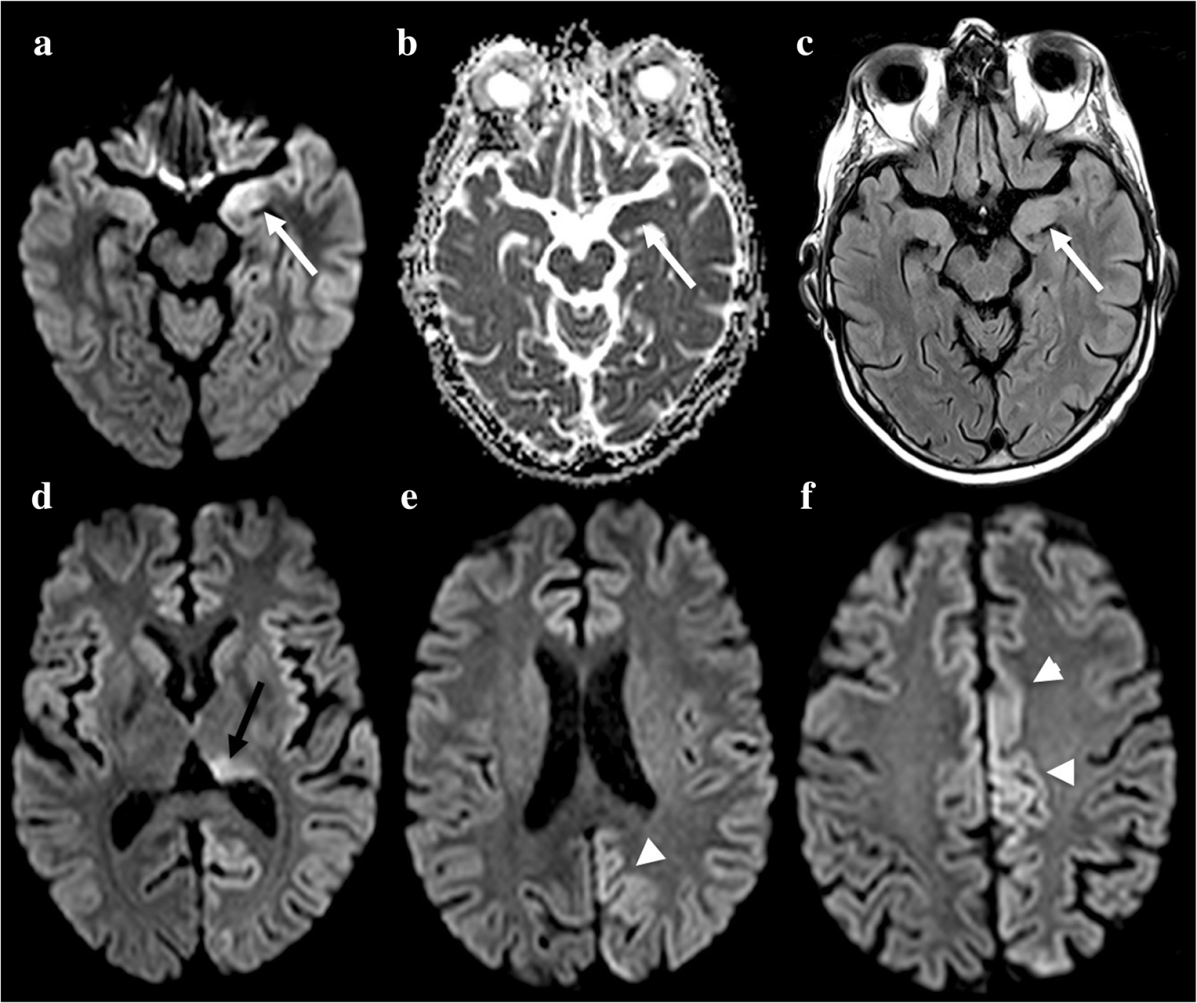
A 50-year-old man with status epilepticus presenting with aphasia and right hemiparesis in a context of chronic alcoholism. Hyperintensities on DWI (**a**, **d**, **e**, **f**) and FLAIR (**c**) with subtle restriction on ADC (**b**) of the left hippocampus (white arrow), pulvinar (black arrow) and cortex (arrowheads)

In the case of a simple epileptic seizure, MRI changes can be focal, multifocal, hemispheric or diffuse [20], but there may also be no changes at all. In status epilepticus, which corresponds to a prolonged series of seizures of at least 20–30 min, during which the patient does not completely regain consciousness or does not exhibit normalisation of his/her EEG, MRI often shows cortical abnormal signal intensities, sometimes associated with pulvinar [21] and hippocampal lesions (Fig. 4). Epileptic activity may be responsible for regional vasogenic and cytotoxic oedema, reflecting haemodynamic and metabolic changes, respectively. This is why the lesions are usually hyperintense on DWI, with no arterial distribution and with variable ADC values. Similarly, they are associated with high signal intensity on FLAIR and T2-WI, low signal intensity on T1-WI and additional cortical swelling. TOF-MRA can reveal prominent arteries facing cortical lesions. PWI may, therefore, show mild hyperperfusion in the epileptic region [22] or be normal. Gyral and leptomeningeal contrast enhancement [23], certainly related to alteration of the leptomeningeal blood–brain barrier, is observed on contrast-enhanced T1-WI. Typically, all these changes lack arterial distribution and are topographically compatible with a clinical seizure or EEG findings because they are on the same side of the periodic discharges. Radiologists must strive to look for an origin of the epilepsy on MRI, such as a tumour or a stroke sequel. Signal changes are usually reversible, but the MRI follow-up may highlight irreversible changes, such as brain atrophy, cortical laminar necrosis and mesial temporal sclerosis, especially in generalised convulsive status epilepticus [24].

### Hypoglycaemia

**Fig. 5:**
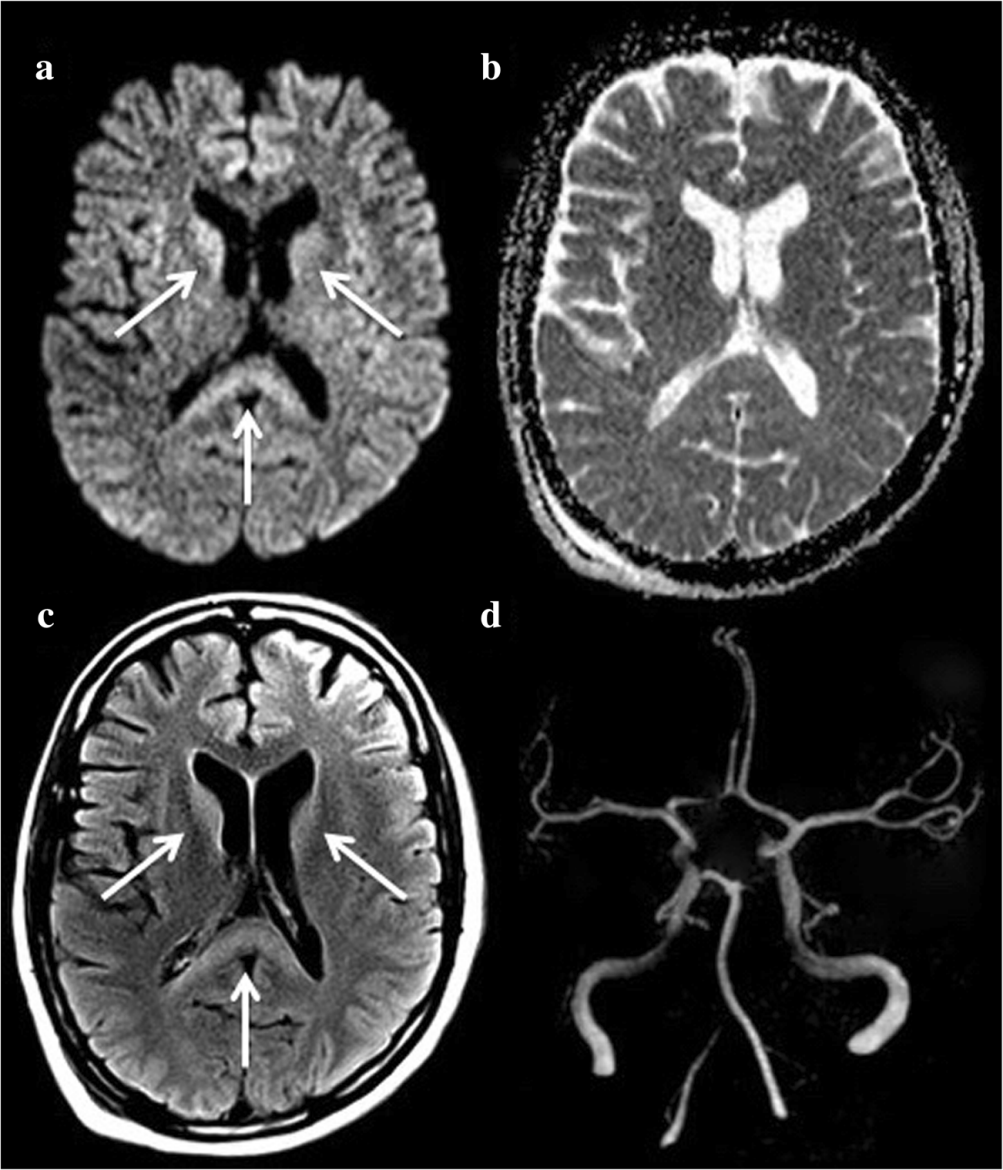
A 42-year-old woman with hypoglycaemia presenting with disturbance of consciousness after abdominal surgery. DWI shows hyperintensities in the basal ganglia and the splenium of the corpus callosum (**a**, **c**, arrows) with no restriction (**b**) and no arterial occlusion (**d**)

Hypoglycaemia, defined by a plasma glucose < 2.5 mmol/L (45 mg/dl), can manifest with various neurologic deficits, such as drowsiness, mood swings, seizures, confusion and coma, but also acute focal deficits, such as hemiplegia, that can be difficult to differentiate from ischaemic stroke. The main cause of hypoglycaemia is the accidental or deliberate overuse of hypoglycaemic agents in a known diabetic patient. Hypoglycaemia may also be induced by severe sepsis, renal or hepatic failure, Addison’s disease or insulin-secreting tumours. MRI may be normal or may show extensive, bilateral and symmetrical, white and grey matter lesions. They then predominate in the white matter (corona radiata, internal capsule and splenium of corpus callosum), and the grey matter areas most often affected are the occipital and temporal lobes, basal ganglia and hippocampi (Fig. 5). The thalamus, hypothalamus, brain stem and cerebellum are generally spared.

DWI is the most sensitive sequence for detecting such lesions by showing decreased ADC, before T2 and FLAIR hyperintensities become visible. The outcome of hypoglycaemic encephalopathy depends on the severity and duration of the hypoglycaemia, but also on the extent and location of brain lesions observed on DWI [25]. Two patterns of lesions exist on DWI [25]: on the one hand, lesions located anywhere along the corticospinal tracts, including the motor cortex, corona radiate, posterior limb of the internal capsule, pyramidal tracts, splenium of the corpus callosum or middle cerebellar peduncles, which are associated with a good clinical outcome, and on the other hand, lesions involving the cerebral cortex away from the motor cortex, basal ganglia or hippocampus, which are associated with a poor prognosis. The prognosis is usually also poor if the lesions do not regress on follow-up [26]. It is important to note that these hyperintense lesions on DWI also appear hyperintense on T2-WI or FLAIR images, even during the acute phase, and are not associated with abnormal findings on PWI. They may demonstrate enhancement on post-gadolinium T1-WI. Magnetic resonance spectroscopy reveals unspecific mildly reduced N-acetylaspartate and no lactate peak in this metabolic disorder.

### MELAS

Mitochondrial myopathy, encephalopathy, lactic acidosis, stroke-like episodes (MELAS) is a rare, multisystem disorder affecting a young population. It belongs to a group of mitochondrial metabolic diseases [27]. This syndrome is caused by an absence or deficit of subunits of the respiratory chain protein complex due to a mutation in mitochondrial DNA [28] and leads to the impaired function of cells or even death. The clinical diagnosis is based on the following features: stroke-like episodes occurring before the age of 40 years, encephalopathy with seizures and/or dementia, the presence of lactic acidosis, ragged red muscle fibres, as well as additional criteria, such as recurrent headaches and recurrent vomiting [27].

The most characteristic neurological features of MELAS are the stroke-like episodes, such as hemiparesis, hemianopsia or cortical blindness. Their pathophysiology is still controversial. Various hypotheses have been proposed: (1) ischaemic vascular mechanism, (2) generalised cytopathic mechanism and (3) non-ischaemic neurovascular cellular mechanism. Because neurological episodes in MELAS mimic ischaemic stroke clinically, MRI is particularly helpful to distinguish them.

**Fig. 6:**
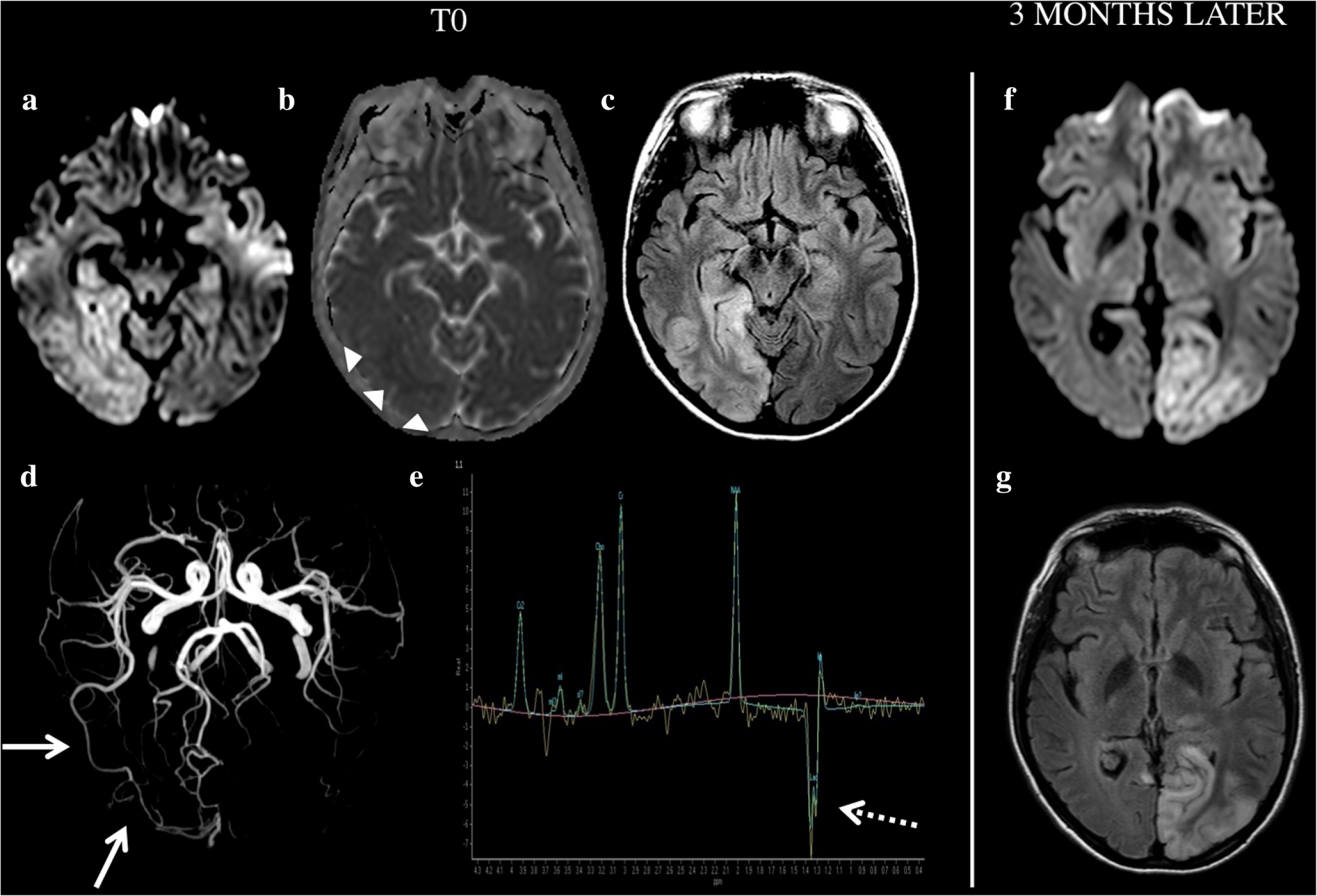
A 19-year-old woman with mitochondrial myopathy, encephalopathy, lactic acidosis, stroke-like episodes (MELAS) presenting with left homonymous hemianopsia. Hyperintensity on DWI (**a**) and FLAIR (**c**) with subtle restriction on ADC (**b**, arrowheads). Time-of-flight (TOF) images show regional arterial vasodilatation (**d**, arrows). Magnetic resonance spectroscopy shows significantly elevated lactate peak at 1.3 ppm (dotted arrow) and decreased NAA peak in the normal-appearing brain. A new lesion appeared 3 months later (**f**, **g**); the patient presented with right homonymous hemianopsia

The locations of stroke-like lesions are correlated with focal neurological symptoms. Such lesions involve the cortex and appear with DWI hyperintensity, as is the case for real ischaemic lesions. Interestingly, the ADC is variable, with a possible mix of increase and decrease [29]. Increased-ADC lesions will regress at follow-up, whereas decreased-ADC portions will persist. Furthermore, the distribution of stroke-like lesions does not follow vascular territories (Fig. 6) and often shows a slow, progressive spread. FLAIR or T2-WI shows cortex swelling and areas of abnormally high signal with loss of cortico-subcortical differentiation. Abnormal cortical veins appearing hyperintense on FLAIR can be seen and may be a reflection of cortical venous stenosis, congestion and venous ischaemia [30]. Contrary to ischaemic stroke, TOF-MRA and PWI can reveal arterial vasodilatation and hyperperfusion in the stroke-like lesions of MELAS. Magnetic resonance spectroscopy may aid in the diagnosis, revealing a significantly elevated lactate peak at 1.3 ppm and decreased NAA, in stroke-like lesions as well as in the normal-appearing brain parenchyma [31].

Finally, stroke-like episodes may recur, but then lesions tend to appear in different cerebral locations.

### PRES

Posterior reversible encephalopathy syndrome (PRES) is a clinico-radiological syndrome characterised by a variable combination of impaired consciousness, seizure activity, headaches, visual abnormalities, nausea/vomiting and focal neurological signs [32]. Prompt diagnosis and the empirical modification of all identifiable risk factors are the determinants of the outcome of patients. The causes are numerous, the most common of which are hypertension, eclampsia and immunosuppressant. The mechanism of PRES is not known. Two opposing hypotheses are commonly cited: (1) the older theory suggests that severe hypertension exceeds the limits of autoregulation, resulting in a brain oedema; (2) the earlier theory suggests that systemic toxicity leads to endothelial dysfunction with subsequent vasoconstriction or leukocyte trafficking, or both [33].

**Fig. 7:**
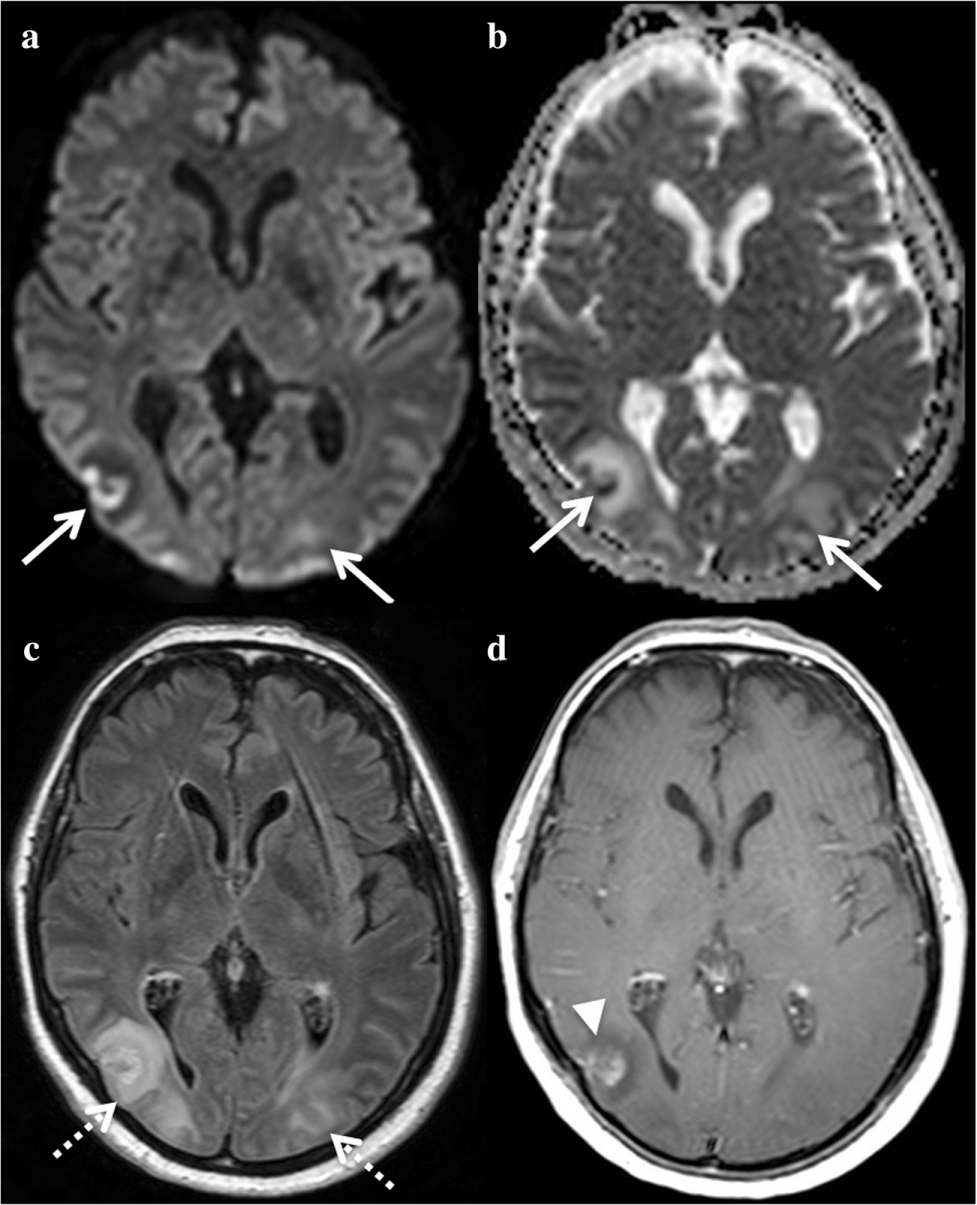
A 40-year-old woman with posterior reversible encephalopathy syndrome (PRES) presenting with postpartum attention deficit disorder. DWI shows both vasogenic and cytotoxic oedema with increased and decreased ADC (**a**, **b**, arrows). Posterior lesions appear as hyperintensities on FLAIR (**c**, dotted arrows). Contrast-enhanced T1-WI shows cortical enhancement of one lesion (**d**, arrowhead)

PRES lesions affect the cortex and subcortical white matter, are hyperintense on T2 and FLAIR, and have a predilection for posterior regions with a suggestive symmetric distribution (Fig. 7). Focal areas of vasogenic oedema may also be seen in the brain stem [34], the basal ganglia and deep white matter (external/internal capsule) [35]. However, focal areas of cytotoxic oedema with restricted diffusion can be seen and mixed patterns on DWI are not uncommon [36]; cytotoxic oedema may be associated with a poor outcome [37]. T2-GRE or SWI can detect subarachnoid haemorrhaging at the convexity or intraparenchymal haematoma. Contrast-enhanced T1-WI can show enhancement of lesions, which is seen more commonly in children than in the adults [38]. TOF-MRA may show features resembling vasculopathy, with focal vasoconstriction/vasodilatation or diffuse vasoconstriction.

### Brain tumour

Patients with brain tumours may initially present with acute focal neurologic symptoms and may mimic a stroke [39]. It is important to distinguish brain tumours from strokes early, to avoid improper treatment such as thrombolytic therapy with a risk of haemorrhage, and not to delay correct management of the brain tumour.

MRI plays a crucial role in the initial diagnosis of brain tumours, treatment planning and in their monitoring.

**Fig. 8:**
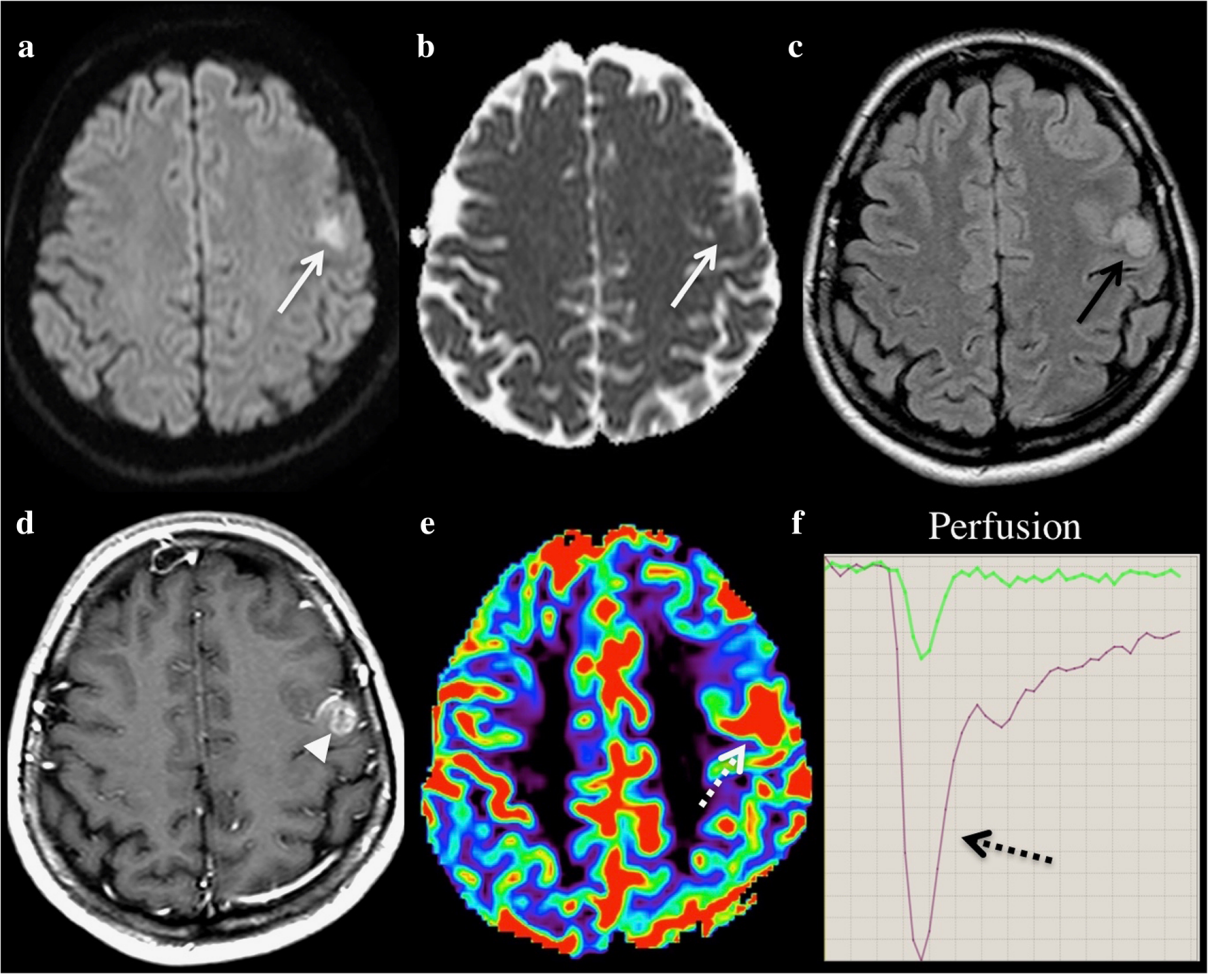
A 54-year-old woman with brain tumour (glioblastoma) presenting with language deficit. DWI shows hyperintensity (**a**, white arrow) with subtle restriction on ADC (**b**, white arrow). Intra-axial left frontal mass with hypersignal FLAIR swelling (**c**, black arrow) and contrast enhancement (**d**, white arrowhead). Perfusion-weighted imaging (PWI) shows increased cerebral blood volume (CBV) (**e**, **f**, dotted arrows)

Conventional T1-WI pre- and post-contrast, T2-WI and FLAIR sequences are usually straightforward and easily distinguish brain neoplasm from AIS by showing a round or ovoid enhancing lesion (Fig. 8) surrounded by vasogenic oedema, which are features not encountered in the first few hours following stroke onset. However, brain tumours may not harbour all these signs and display as a stroke mimic on the initial MRI. This can happen especially if tumours are small, included in an arterial territory and involve the cortex.

DWI shows variable signal intensities for primary and secondary tumours. The ADC is inversely proportional to cellular density. Therefore, the ADC value of high-grade gliomas is classically lower than that of low-grade gliomas [40]. In the same way, lymphoma tends to have low ADC due to its high cellularity. The tumour may, therefore, appear with high DWI signal intensity. PWI may then be helpful because solid tumours do not show areas of low relative cerebral blood volume (rCBV) as a stroke would. On the contrary, rCBV tends to increase with neoplasm grade [41]. Magnetic resonance spectroscopy may be useful too, as the typical pattern of an intra-axial tumour includes an elevated peak of choline and reduced NAA, the former not being present in an acute stroke. In cases where the diagnosis remains uncertain, follow-up will confirm or deny the presence of a brain tumour.

### Demyelinating diseases

Multiple sclerosis is the leading cause of non-traumatic neurological disability in young adults. In 85% of patients, multiple sclerosis follows a relapsing–remitting course and is associated with acute demyelinating lesions [42].

On DWI, these acute demyelinating lesions sometimes demonstrate hyperintensity, generally associated with an increased ADC. However, acute demyelinating lesions have also been described with a decreased ADC [43], which raises the issue of differential diagnosis with ischaemic stroke, especially if no contrast-enhanced MRI is performed. It is important to note that most acute demyelinating lesions only involve cerebral white matter and do not demonstrate cortico-subcortical arterial distribution. T2-WI or FLAIR shows multiple white matter lesions, which might be disseminated in space and time according to the McDonald criteria [44], to be diagnosed as multiple sclerosis. Dissemination of lesions in space is demonstrated by at least one T2 lesion in at least two of the four following areas of the central nervous system: (1) periventricular, (2) juxtacortical, (3) infratentorial and (4) spinal cord. The European collaborative research network that studies MRI in MS (MAGNIMS) proposed new MRI criteria to be applied in multiple sclerosis, adding optic nerve lesions as an additional area to the dissemination in space, increasing the number of DIS locations from 4 to 5 [45]. Note that this area may not be conserved in the future McDonald criteria.

**Fig. 9:**
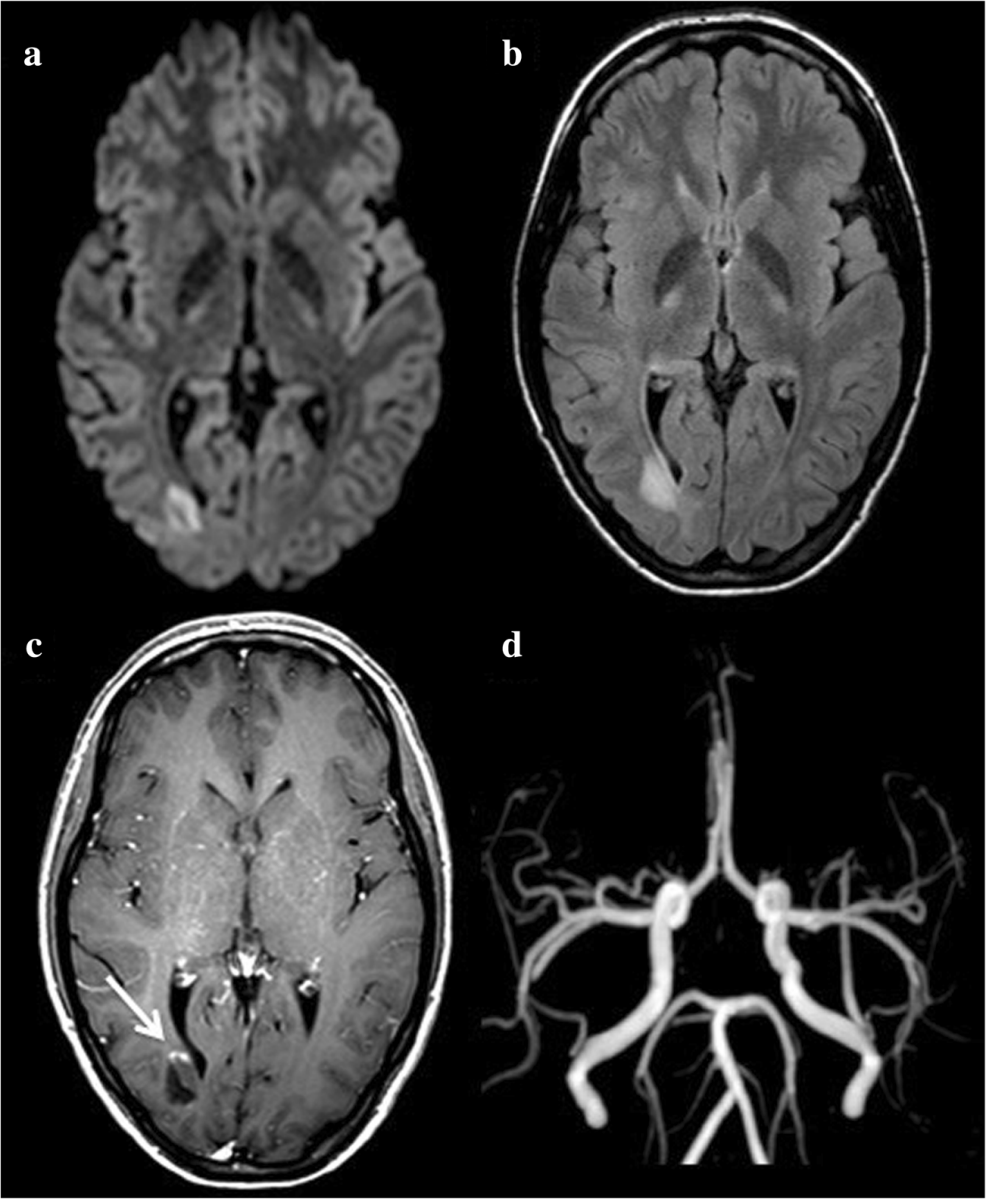
A 17-year-old woman with multiple sclerosis presenting with left homonymous hemianopsia. DWI shows ring hyperintensity (**a**). The lesion appears as hyperintensity on FLAIR (**b**) with gadolinium enhancing (**c**, arrow). No arterial occlusion (**d**)

Additionally, contrast-enhanced T1-WI may highlight gadolinium uptake in acute lesions (Fig. 9), a feature not encountered in the first few hours following stroke onset. Furthermore, the simultaneous presence of asymptomatic gadolinium-enhancing and non-enhancing lesions on a single MRI now proves dissemination of lesions in time based on the McDonald criteria. PWI is not really helpful because both mildly increased [46] and decreased [47] perfusions have been reported. Magnetic resonance spectroscopy may instead help diagnose acute demyelinating lesions as it shows a reduction of the N-acetylaspartate peak while choline is typically increased [58], and a negative doublet corresponding to lactate is abnormally visible on long TE [49].

### Susac’s syndrome

Susac’s syndrome is a form of retinocochleocerebral arteriopathy. There is a strong predilection in women between the ages of 20 and 40 years. Susac’s syndrome is probably caused by the obstruction of pre-capillary arterioles of the brain, retina and inner ear, secondary to lesions from anti-endothelial cell antibodies [50]. The physiopathology of the selective tissue distribution is still not clearly understood; this could be explained by a common embryologic origin of the brain [51]. Symptoms include visual and hearing disturbances, as well as neurological deficits, the latter mimicking stroke or transient ischaemic attacks. Susac’s syndrome needs to be treated early, aggressively and durably to prevent relapses. Although the obstruction of arterioles is responsible for microinfarcts, treatment is not covered by thrombolysis but by immunosuppressant drugs [52], based on the hypothesis of being an autoimmune disease.

**Fig. 10:**
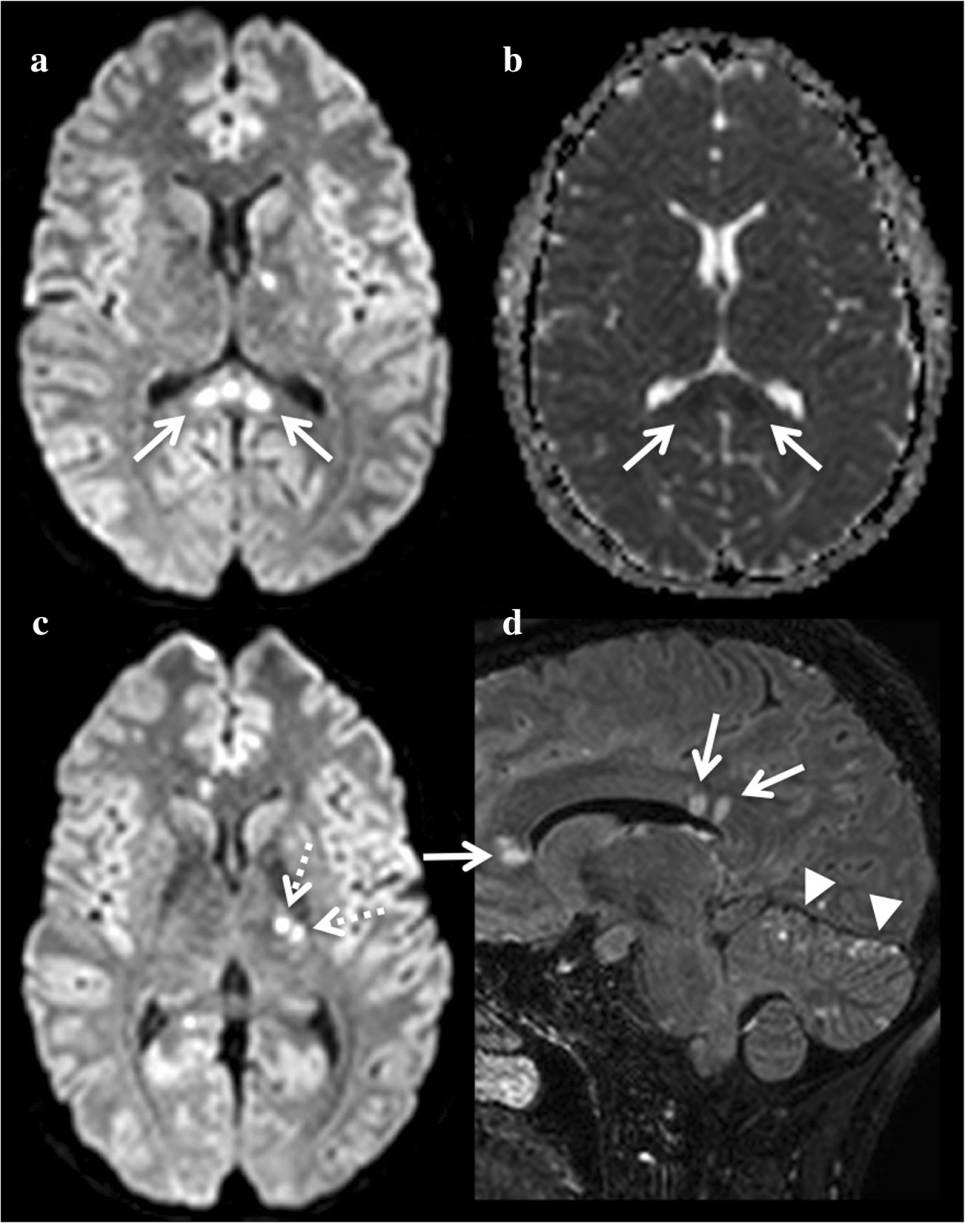
A 25-year-old woman with Susac’s syndrome presenting with vertigo and vertical diplopia. DWI shows restrictive lesions in the centre of the corpus callosum described as ‘snowballs’ (**a**, **b**, arrows) and in the posterior limb of internal capsule appearing as a ‘string of pearls’ (**c**, dotted arrows). Contrast-enhanced FLAIR shows small round white matter lesions (‘snowballs’) in the central fibres of the corpus callosum (**d**, arrows) and leptomeningeal contrast enhancement predominant in the posterior cranial fossa (**d**, arrowheads)

On MRI, acute lesions appear as small multifocal lesions of 3–7 mm spread in both white matter and basal ganglia, typically demonstrating DWI and FLAIR high signal intensity. A more specific pattern is described on DWI that shows restrictive lesions in the centre of the corpus callosum, described as ‘snowballs’, and in the posterior limb of internal capsule appearing as a ‘string of pearls’ (Fig. 10). The central location of the lesions of the corpus callosum is important to differentiate Susac’s syndrome from multiple sclerosis, in which lesions are more readily at the under-surface and at the septal interface of the corpus callosum. The combination of typical central callosal lesions and a ‘string of pearls’ in the internal capsule is considered pathognomonic for Susac’s syndrome [53]. Contrast-enhanced T1-WI can highlight suggestive leptomeningeal contrast enhancement [54]. Three-dimensional TOF is usually normal because Susac’s syndrome affects only the precapillary arterioles, which are below the resolution of magnetic resonance angiography.

### Herpes simplex encephalitis

**Fig. 11:**
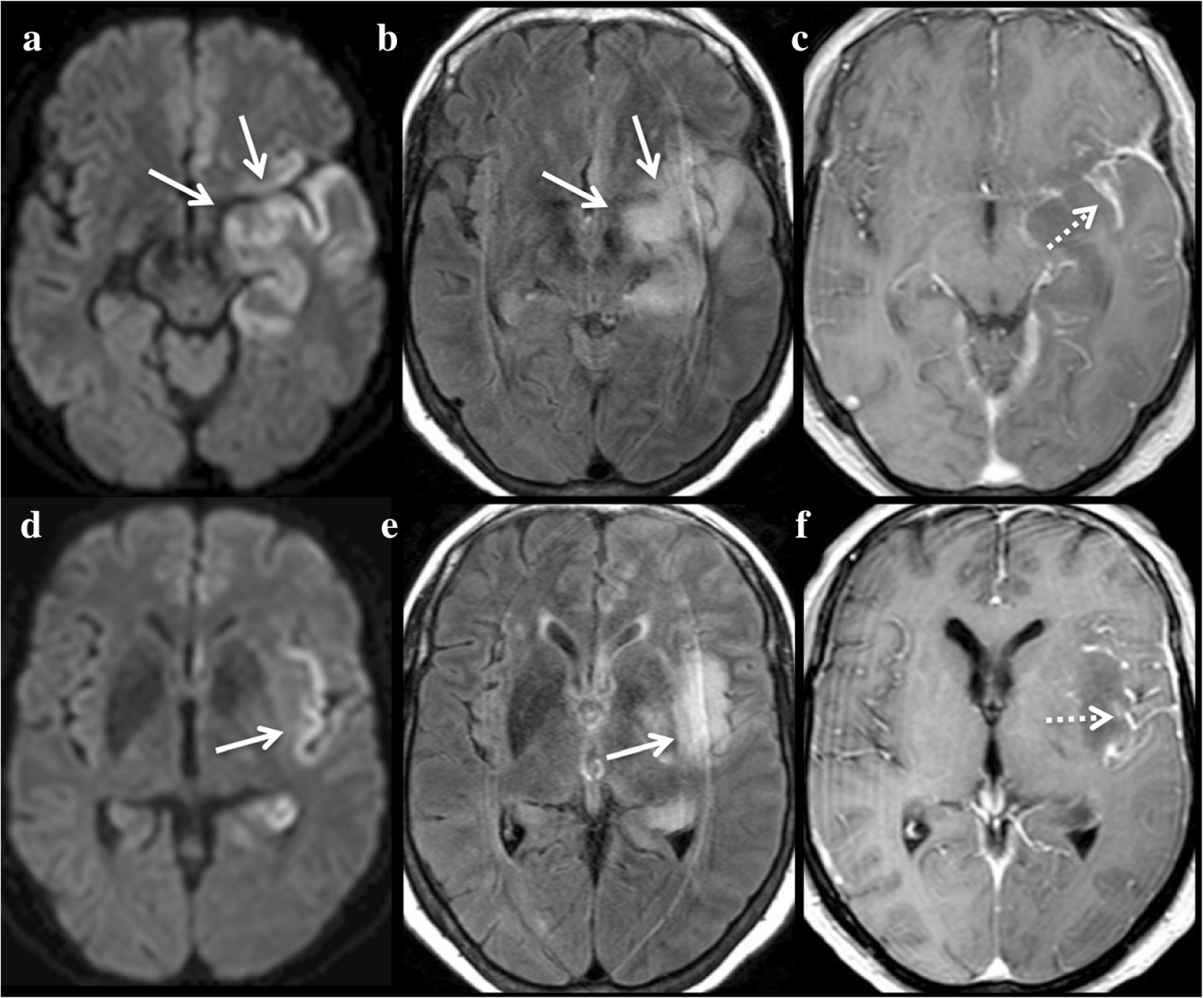
A 63-year-old woman with herpes encephalitis presenting with aphasia, cephalalgia and hyperthermia. DWI and FLAIR show left temporal and insular hyperintensities involving the uncus (**a**, **b**, **d**, **e**, arrows). Contrast-enhanced T1-WI shows leptomeningeal enhancement related to the associated meningitis (**c**, **f**, dotted arrows)

Herpes simplex virus (HSV) encephalitis is the most common cause of fatal sporadic fulminant necrotising viral encephalitis, usually due to HSV-1 in most patients and HSV-2 in the remainder. HSV encephalitis manifests as a bilateral asymmetrical involvement of the limbic system, medial temporal lobes, insular cortices and inferolateral frontal lobes. The basal ganglia are typically spared, helping to distinguish it from a middle cerebral artery stroke. DWI is known to be more sensitive than T2-weighted images to highlight the parenchymal lesions and shows restricted diffusion due to cytotoxic oedema at the acute phase. A lesion involving only a single medial temporal lobe could be mistaken for a middle cerebral artery stroke; in that case, the anterior aspect of the parahippocampal gyrus, called the uncus, is spared, this small area being supplied by the anterior choroidal artery (Fig. 11). The lesions are hyperintense on T2-WI and FLAIR. T2-GRE or SWI may demonstrate blooming if the HSV encephalitis is haemorrhagic. Additionally, contrast-enhanced T1-WI may highlight gadolinium uptake with gyral enhancement due to the breakdown of the blood–brain barrier or with leptomeningeal enhancement related to the associated meningitis.

## Stroke mimics with normal DWI

### Migraine aura

Migraines are a frequent pathology affecting 17.1% of women and 5.6% of men in the United States [55]. Less than 30% of migraine attacks are preceded by an aura, which is defined as any negative or positive neurologic symptom that usually lasts less than 60 min. The pathophysiology of migraine aura is still debated, with two existing and opposing theories. The vascular theory is based on the idea that vasoconstriction/hypoperfusion is associated to the aura and vasodilatation/hyperperfusion to the headache. The other theory suggests that a contiguous wave of neuronal depression spreads through the cortex, causing the aura symptoms [56]. Migraines with aura typically present with acute neurologic deficit, thus mimicking AIS, especially when the aura occurs with minimal or no headache.

**Fig. 12:**
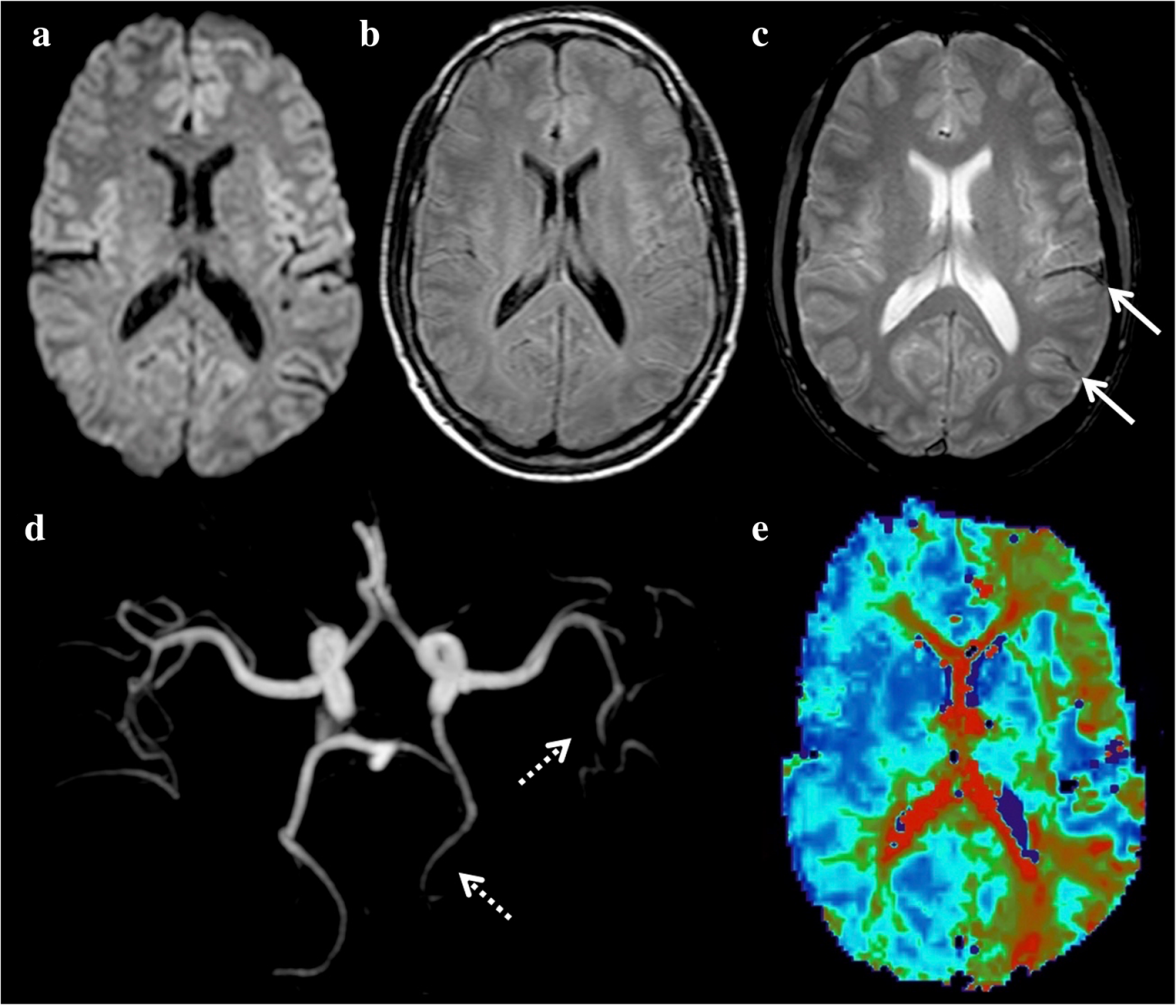
A 38-year-old man with migraine aura presenting with motor, sensory and language deficits. DWI shows no hyperintensity (**a**). FLAIR is unremarkable (**b**). Hypointense signals of the venous on T2* (**c**, arrows). Regional and distal spasm of the arteries on TOF-MRA (**d**, dotted arrows) in relation with the hypoperfused areas with delayed TTP (**e**)

MRI is useful for early distinction between a migrainous aura and a stroke, as, in the former, PWI demonstrates focal cerebral hypoperfusion not distributed to arterial territories (Fig. 12). Though DWI, T1-WI, T2-WI and FLAIR are unremarkable in acute migrainous aura, PWI demonstrates hypoperfusion in up to 70% of cases [57]. Such hypoperfusion in the aura presents with a suggestive pattern including delayed MTT and TTP, decreased CBF and minimal decreased CBV [67]. Unlike ischaemic stroke, perfusion defects affect more than one arterial territory in a migrainous aura. These abnormalities are observed in brain regions corresponding to the patient’s symptoms, with a predilection for the posterior regions. Bilateral hypoperfusion can be seen, and TOF-MRA may reveal regional and distal loss of visibility of the arteries in relation with the hypoperfused areas [58]. On the other hand, because of the increased oxygen extraction and relative increase of deoxyhaemoglobin in the hypoperfused brain parenchyma, T2-GRE or SWI shows dilated cortical veins draining these regions [59]. It is important to note that some studies report hyperperfusion in migraines, but distant to the aura, during the headache stage, when diagnosis is then more obvious [60].

### HaNDL

Headaches with associated neurological deficits and lymphocytosis (HaNDL) are characterised by temporary recurrent neurological deficits, moderate to severe headache, cerebrospinal fluid lymphocytosis, elevated protein and increased opening pressure. Migraine aura and HaNDL may share a common pathophysiological pathway, resulting in similar imaging findings, namely, no altered diffusion-weighted images, no arterial occlusion, but hypoperfusion in more than one arterial territory [61].

**Fig. 13:**
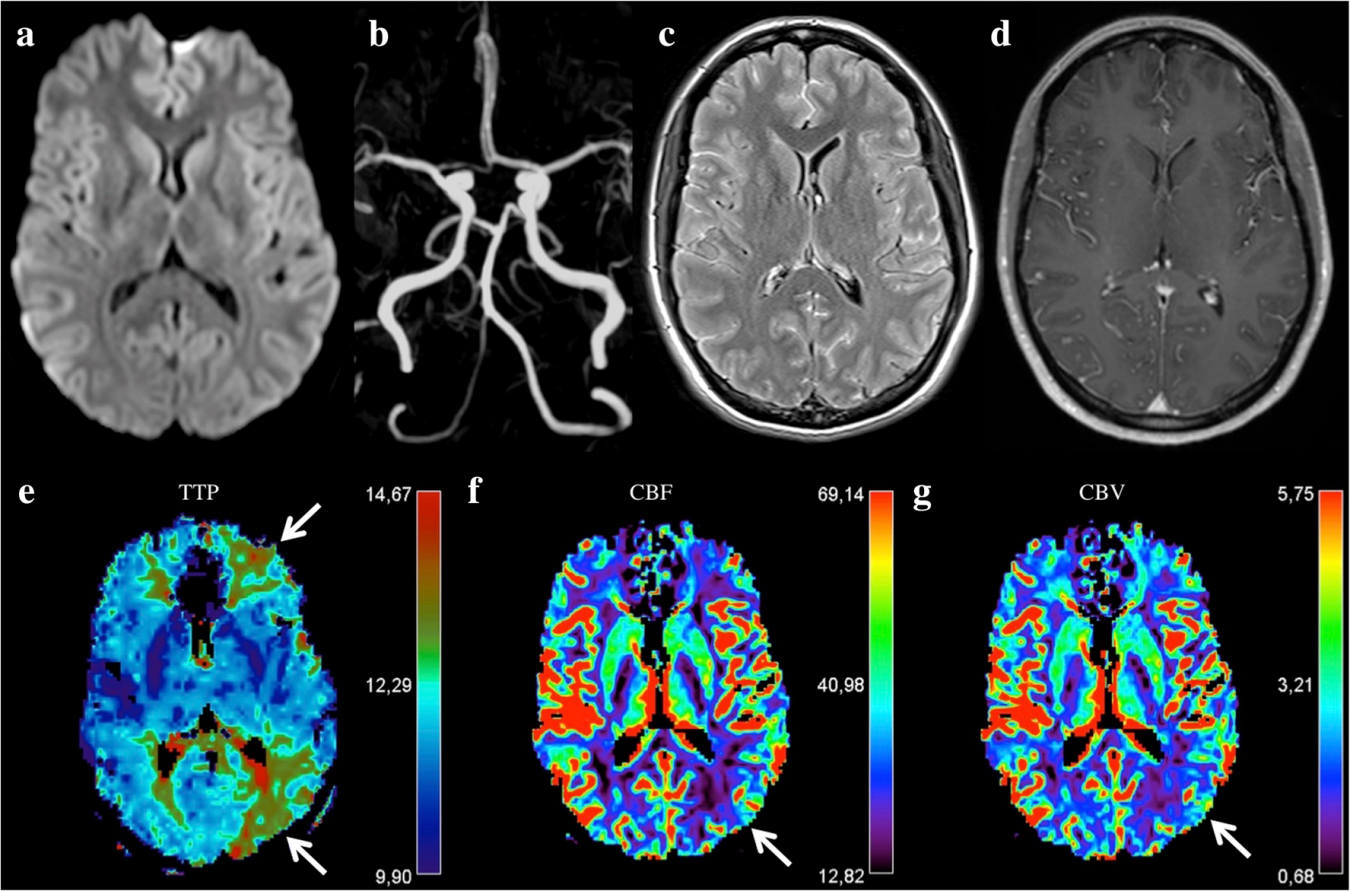
A 61-year-old woman with headaches with associated neurological deficits and lymphocytosis (HaNDL) presenting with motor and language deficits. No altered DWI (**a**), no arterial occlusion (**b**). Hyperintensities on FLAIR (**c**) and enhancement (**d**) in the cortical sulci may be seen related to lymphocytic meningitis. Hypoperfusion can be seen in more than one arterial territory (**e**–**g**, arrows)

Additionally, leptomeningeal hyperintensity on FLAIR images with corresponding enhancement on post-contrast T1-WI, certainly related to lymphocytic meningitis, are suggestive MRI features of HaNDL in this clinical setting (Fig. 13). Of course, HaNDL remains a diagnosis of exclusion, but MRI is useful for early distinction between this syndrome and a stroke.

### Cortical subarachnoid haemorrhage in cerebral amyloid angiopathy

Cerebral amyloid angiopathy (CAA) is characterised by the progressive deposition of amyloid-β in the wall of cortical and leptomeningeal arteries [62]. CAA is associated with transient focal neurological episodes (TFNE), sometimes termed ‘amyloid spells’. These include both positive and negative neurological symptoms, and may be caused by convexity subarachnoid haemorrhaging. Such acute subarachnoid haemorrhage in CAA is usually limited to a few sulci at the convexity of the brain, but predicts a high early risk of symptomatic intracerebral haemorrhage, which may motivate preventive action [63]. These TFNE are mostly recurrent, stereotyped and brief. The most common negative symptom is spreading brachiofacial paresia [64]. It is important to note that these symptoms are usually not associated with sudden headaches, even if they are related to an acute subarachnoid haemorrhage.

**Fig. 14:**
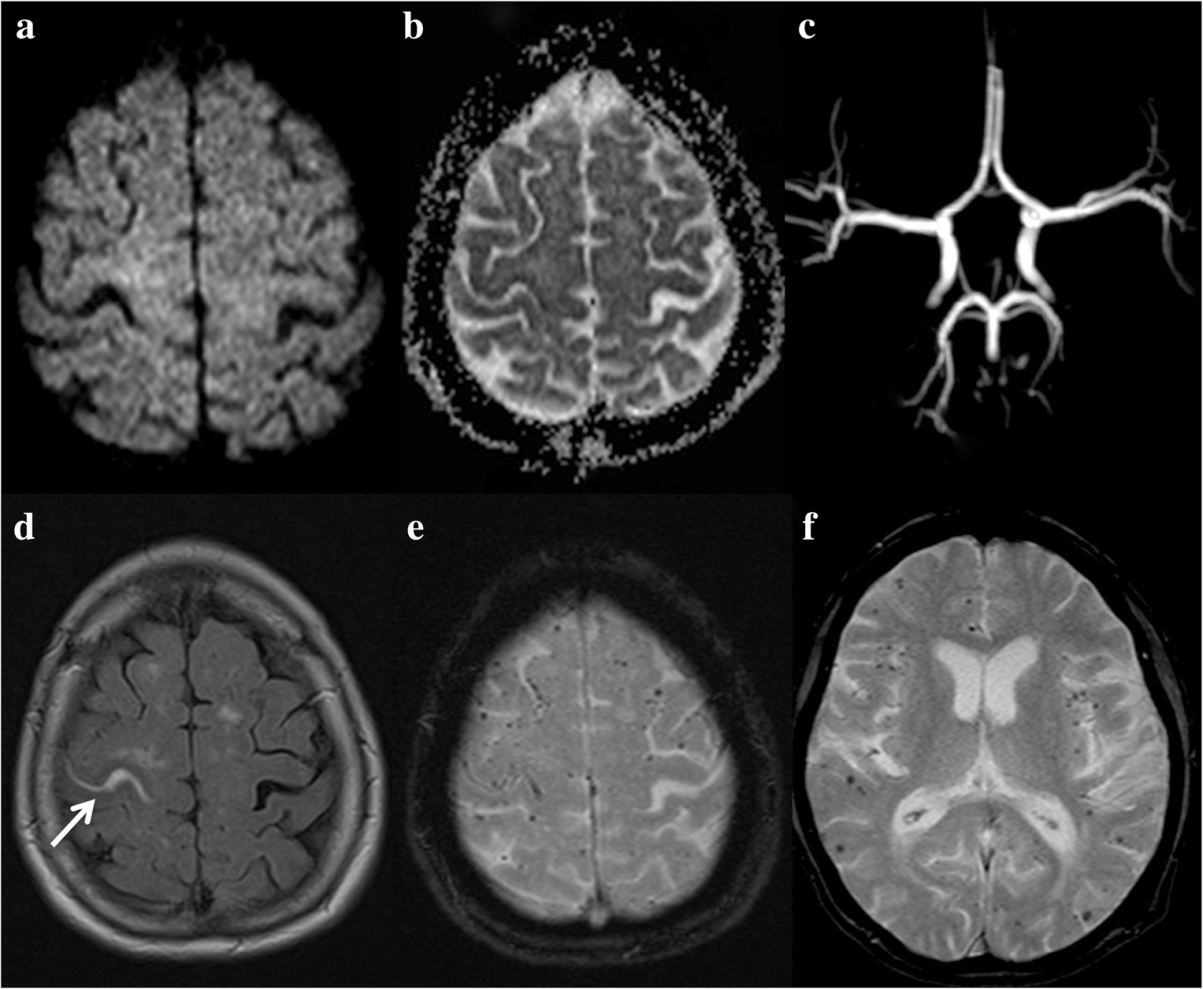
A 68-year-old woman with cerebral convexity subarachnoid haemorrhage (cSAH) in cerebral amyloid angiopathy (CAA) presenting with paraesthesia of the left upper extremity and of the left side of the face. DWI shows no hyperintensity (**a**) and no restriction (**b**). No arterial occlusion (**c**). FLAIR shows hyperintensity in cortical sulcus (**d**, arrow). T2-GRE shows microbleeds that spared the basal ganglia (**e**, **f**)

MRI depicts acute cerebral convexity subarachnoid haemorrhage (cSAH) in a few sulci with a predilection for the central sulcus [65]. Such acute cSAH appears hyperintense on FLAIR images (Fig. 14), hypointense on T2-GRE images and presents with focal enhancement on post-contrast T1-WI. No acute parenchymal abnormalities are observed on MRI, especially on DWI. TOF-MRA is normal too. White matter T2 hyperintensities and chronic haemorrhages responding to Boston criteria in CAA are observed on T2-GRE images, namely cortical superficial siderosis, microbleeds or intracerebral haematoma [66]. The multifocality of cortical superficial siderosis correlates with disease severity in CAA [67].

If acute non-traumatic cSAH is isolated, other diagnoses must be sought, such as arteriovenous malformations or fistulas, cortical vein or dural sinus thrombosis, or distal or proximal arteriopathies, and should trigger a complete non-invasive imaging protocol, including parenchymal and vascular magnetic resonance imaging [68].

## Conclusion

Differentiating a real ischaemic stroke from a stroke mimic a few hours after the onset of neurological symptoms can be a challenge for both neurologists and radiologists. Magnetic resonance imaging (MRI) is the best examination to demonstrate early signs of ischaemia, but also allows for the recognition of stroke mimics, thus optimising their adequate management.

The practical illustrated algorithm of the main ‘stroke mimics’ we propose here, based on the results of diffusion-weighted imaging (DWI) and common additional magnetic resonance sequences, hopefully provides enough confidence to evoke such alternative diagnoses (Fig. 1).
